# Final analysis results from DREAMM-3, a phase 3 study of belantamab mafodotin versus pomalidomide plus dexamethasone in patients with relapsed/refractory multiple myeloma

**DOI:** 10.1038/s41408-026-01646-0

**Published:** 2026-10-17

**Authors:** Meletios Athanasios Dimopoulos, Vania Hungria, Haitao Meng, Atanas Radinoff, Jian Li, Sosana Delimpasi, Ting Niu, Gabor Mikala, Tamas Masszi, Marcelo Capra, Michael Low, Angelo Maiolino, Yan Xu, Xavier Leleu, Vasiliki Pappa, Dominik Chraniuk, Tatiana Konstantinova, Iurii Osipov, Shawn Hakim, Chris Brawley, Gabriela Tubel, Astrid McKeown, Julia Boyle, Sumita Roy-Ghanta, Katja Weisel

**Affiliations:** 1 National and Kapodistrian University of Athens, Athens, Greece; 2 Korea University, Seoul, South Korea; 3 Department of Hematology, Clinica São Germano São Paulo, São Paulo, Brazil; 4 The First Affiliated Hospital, Zhejiang University School of Medicine, Hangzhou, China; 5 University Hospital “St Ivan Rilski” EAD, Sofia, Bulgaria; 6 Department of Hematology, Peking Union Medical College Hospital, Chinese Academy of Medical Sciences and Peking Union Medical College, Beijing, People’s Republic of China; 7 General Hospital Evangelismos, Athens, Greece; 8 West China Hospital of Sichuan University, Chengdu, Sichuan China; 9 South Pest Central Hospital, National Institute for Haematology and Infectious Diseases, Budapest, Hungary; 10 Semmelweis University Department of Internal Medicine and Hematology, Budapest, Hungary; 11 Centro Gaúcho Integrado de Pesquisa, Hospital Mãe de Deus, Porto Alegre, Brazil; 12 Monash Haematology, Monash University, Clayton University, Victoria, Australia; 13 Instituto Americas de Ensino, Pesquisa e Inovacao, Rio de Janeiro, Brazil; 14 Universidade Federal do Rio de Janeiro, Rio de Janeiro, Brazil; 15 Institute of Hematology and Blood Disease Hospital, Chinese Academy of Medical Sciences, Tianjin, China; 16 Hospital of Poitiers, Poitiers, France; 17 Second Department of Internal Medicine and Research Unit, Hematology Unit, University General Hospital “Attikon”, Athens, Greece; 18 Department of Hematology, Wojewodzki Szpital Zespolony, Torun, Poland; 19 Regional Clinical Hospital No 1, Ekaterinburg, Russian Federation; 20 V.A. Almazov National Medical Research Centre, Saint-Petersburg, Russian Federation; 21 GSK, Upper Providence, PA USA; 22 GSK, London, UK; 23 GSK, Stevenage, UK; 24 University Medical Center of Hamburg-Eppendorf, Hamburg, Germany

**Keywords:** Myeloma, Targeted therapies

**To the Editor**,

Targeting B-cell maturation antigen (BCMA) has transformed the relapsed/refractory multiple myeloma (RRMM) landscape with unprecedented overall survival (OS) outcomes versus standard-of-care triplet regimens. Belantamab mafodotin, an anti-BCMA antibody-drug conjugate, has a multimodal mechanism of action involving minimal immunosuppression and no evidence of induction of T-cell exhaustion [1, 2]. DREAMM-3 (NCT04162210) was a confirmatory phase 3 study to support the initial United States Food and Drug Administration and European Medicines Agency authorization of belantamab mafodotin monotherapy for patients with RRMM who had ≥4 prior lines of therapy (LOTs) [3, 4]; approval was withdrawn following DREAMM-3 findings, as the primary endpoint of progression-free survival (PFS) superiority against the doublet comparator, pomalidomide-dexamethasone, was not met [5–7]. Subsequently, belantamab mafodotin combination regimens with bortezomib-dexamethasone (BVd) and pomalidomide-dexamethasone (BPd) have received approval for patients with RRMM who have received ≥1 prior LOT in multiple countries [8]. In the US, BVd was approved for patients with RRMM who have received ≥2 prior LOT [9].

DREAMM-3 post-primary analysis updates showed an improved PFS hazard ratio (HR) (0.90 [95% confidence interval (CI) 0.65–1.24]) [7]. We present the final analysis of DREAMM-3 to inform on the long-term efficacy and safety of belantamab mafodotin monotherapy.

The full methodology for the multicenter, open-label DREAMM-3 trial has been published [7]. Supplementary Figure 1 shows the study design. Briefly, patients were randomized 2:1 to receive single-agent belantamab mafodotin or pomalidomide-dexamethasone until progressive disease, death, unacceptable toxicity, loss to follow-up, or end of study. Full dosing schedules are provided in the Supplementary Methods.

Patients had a confirmed MM diagnosis (International Myeloma Working Group [IMWG] criteria) and received ≥2 prior LOTs (including lenalidomide and proteasome inhibitor).

The primary outcome was investigator-assessed PFS (IMWG criteria) [7]. Secondary efficacy outcomes included OS, overall response rate, clinical benefit rate (comprising ≥minimal response), duration of response (DOR), time to progression, and minimal residual disease negativity rate at 10^-5^ threshold by next-generation sequencing. Secondary safety outcomes included adverse events (AEs) measured by Common Terminology Criteria for Adverse Events (CTCAE) and findings during ophthalmic examinations. Prespecified exploratory endpoints included time to best response (TBR) and time from randomization to progression on subsequent line (PFS2). Updated PFS and OS HR for the intention-to-treat populations are reported. As the primary analysis had already concluded without meeting the superiority threshold, the final analysis was descriptive; no formal hypothesis testing was pre-specified.

The study was conducted in accordance with the Declaration of Helsinki and Council for International Organizations of Medical Sciences International Ethical Guidelines, applicable International Conference on Harmonization Good Clinical Practice Guidelines, and applicable laws and regulations. All protocols and amendments were approved by an institutional review board and independent ethics committee, detailed in the Supplement. All patients provided written informed consent before study enrollment.

In total, 325 patients randomized to the belantamab mafodotin arm (*n* = 218) or the pomalidomide-dexamethasone arm (*n* = 107) were included in the final analysis (April 22, 2025). Patients were a median 68 (38–90) years of age (22% [*n* = 70] were ≥75 years), 57% (*n* = 184) were male, and 78% (*n* = 246) were White. Characteristics were balanced between arms. Full patient demographics have been reported [7]. Median (interquartile range) duration of follow-up was 22.2 (7.0–45.7) and 21.9 (8.4–42.8) months for the belantamab mafodotin and pomalidomide-dexamethasone arms, respectively. Belantamab mafodotin-treated patients received a median of 5 treatment cycles over a median (range) of 4.5 (0.4–54.3) months. For pomalidomide-dexamethasone, patients were treated for a median (range) of 5.5 (0.4–55.2) months.

For belantamab mafodotin versus pomalidomide-dexamethasone, respectively, the median PFS at this analysis was 11.2 (95% CI: 6.5, 14.5) and 7.0 (4.6, 10.6) months (HR [95% CI]: 0.87 [0.64, 1.18]); median OS was 24.0 (95% CI: 19.4, 33.2) and 22.9 (16.0, 29.3) months (HR [95% CI]: 0.98 [0.73, 1.30]); median DOR was 25.3 (95% CI: 20.7, 31.8) and 9.9 (7.6, 20.2) months **(**Supplementary Fig. 2**)**; stringent complete response (9%/3%) and complete response or better with MRD negativity (8%/<1%) rates favored belantamab mafodotin over pomalidomide-dexamethasone (Table 1). Median PFS2 was 17.3 (95% CI: 13.8, 21.7) and 13.0 (10.6, 15.5) months (Table 1).

**Table 1:** Efficacy summary.

	Belantamab mafodotin (*N* = 218)	Pomalidomide-dexamethasone (*N* = 107)
Median PFS*, months	11.2	7.0
95% CI	6.5, 14.5	4.6, 10.6
**HR**	0.87	
95% CI	0.64, 1.18	
Median OS*, months	24.0	22.9
95% CI	19.4, 33.2	16.0, 29.3
**HR**	0.98	
95% CI	0.73, 1.30	
**ORR**^**†**^, ***n* (%)**	90 (41)	38 (36)
95% CI	34.7, 48.1	26.5, 45.4
**sCR**	20 (9)	3 (3)
**CR**	12 (6)	4 (4)
**VGPR**	31 (14)	6 (6)
**PR**	27 (12)	25 (23)
**CBR**^**‡**^, *n*** (%)**	103 (47)	50 (47)
**MRD-**^**§**^ **and CR**+, ***n* (%)**	17 (8)	1 (<1)
95% CI	4.6, 12.2	0.0, 5.1
**Median DOR, months**	25.3	9.9
95% CI	20.7, 31.8	7.6, 20.2
Median TBR*^¶^**, months**	5.6	2.1
IQR	2.8, 10.6	1.4, 7.2
Median TTP*, months	11.3	8.6
95% CI	7.0, 14.6	5.7, 11.8
**HR**	0.99	
95% CI	0.71, 1.38	
Median PFS2**, **months**	17.3	13.0
95% CI	13.8, 21.7	10.6, 15.5

*Time to event outcomes (PFS, OS, DOR, TTP, and PFS2) were calculated using the Kaplan-Meier method, with hazard ratios derived from Cox proportional hazards models.

^†^ORR was defined as the percentage of patients with confirmed ≥PR.

^**‡**^CBR was defined as the percentage of patients with confirmed ≥MR.

^**§**^10^-5^ threshold by next-generation sequencing.

^**¶**^TBR was summarized descriptively by treatment group in patients with confirmed ≥PR as the best overall response. Patients with unknown or missing responses were treated as non-responders.

**Therapies received following the DREAMM-3 trial were not standardized between DREAMM-3 study arms and may confound comparison between study arms.

*CBR* clinical benefit rate, *CI* confidence interval, *CR* complete response, *DOR* duration of response, *HR* hazard ratio, *IQR* interquartile range, *MRD -* minimal residual disease negativity, PFS progression-free survival, PFS2 PFS on subsequent line of therapy, *PR* partial response, *OS* overall survival, *sCR* stringent complete response, *TBR* time to best response, *TTP* time to progression, *VGPR* very good partial response.

Most patients experienced an AE, and ≥66% of patients experienced ≥1 maximum Grade 3–4 AE in both treatment groups (belantamab mafodotin 71%, pomalidomide-dexamethasone 66%) (Table 2). Any grade blood and lymphatic system disorders occurred in both arms (belantamab mafodotin 57%, pomalidomide-dexamethasone 64%), including thrombocytopenia (35%/34%), anemia (29%/34%), and neutropenia (12%/40%). Blurred vision was the most common ocular AE in both treatment groups (belantamab mafodotin 48%, pomalidomide-dexamethasone 7%). Increases in aspartate aminotransferase (17%/6%), gamma-glutamyltransferase (14%/6%), and alanine aminotransferase (9%/10%), and abnormal liver function (6%/0%) were reported in patients treated with belantamab mafodotin/pomalidomide-dexamethasone (Supplementary Table 1). Belantamab mafodotin treatment-emergent AEs were generally managed through dose delays and/or reductions [9]. Exposure-adjusted incidence rates (number of patients with event/total person-years of treatment exposure) for infections/infestations were 51.8 per 100 person-years in patients who received belantamab mafodotin (15.6 Grade 3+) and 62.8 (38.8 Grade 3+) for pomalidomide-dexamethasone.

**Table 2:** Safety summary.

	Belantamab mafodotin	Pomalidomide-dexamethasone
	(*N* = 217)^†^	(*N* = 102)^†^
**Any AEs**	212 (98)	98 (96)
Related to study treatment	188 (87)	79 (77)
Leading to permanent discontinuation of study treatment	36 (17)	21 (21)
Leading to dose reduction	83 (38)	42 (41)
Leading to dose interruption/delay	136 (63)	56 (55)
Related to study treatment and leading to permanent discontinuation of study treatment	12 (6)	5 (5)
Maximum Grade 3 or 4 AEs	154 (71)	67 (66)
Related to study treatment	126 (58)	56 (55)
**Non-ocular AEs affecting ≥ 10% of patients,** ***n*** (%) *		
Blood and lymphatic system disorders	124 (57)	65 (64)
Thrombocytopenia	76 (35)	35 (34)
Anemia	64 (29)	35 (34)
Neutropenia	27 (12)	41 (40)
Leukopenia	9 (4)	10 (10)
Infections and infestations	103 (47)	47 (46)
COVID-19	37 (17)	16 (16)
Pneumonia	12 (6)	12 (12)
**Ocular AEs**	155 (71)	23 (23)
Blurred vision	105 (48)	7 (7)
Dry eye	82 (38)	3 (3)
Foreign body sensation in eyes	80 (37)	5 (5)
Photophobia	63 (29)	4 (4)
Eye irritation	63 (29)	3 (3)
Eye pain	49 (23)	0
Reduced visual acuity	40 (18)	1 (<1)
Keratopathy	22 (10)	1 (<1)
Punctate keratitis	22 (10)	0
**Any SAE,** ***n*** **(%)**	101 (47)	45 (44)
Related to study treatment	26 (12)	12 (12)
Fatal	19 (9)	14 (14)
Related to study treatment	0	1 (<1)

*Additional AEs reported in ≥10% of patients are included in Supplementary Table 1.

^**†**^The safety populations included all randomized patients who had received at least one dose of study treatment.

*AE* adverse event, *SAE* serious adverse event.

Grades for ophthalmic examination findings (OEFs) were investigator-assessed or derived where no investigator-assessed grades were available. Any-grade OEFs were derived by the sponsor in 133/168 (79%) patients who were treated with belantamab mafodotin and who had no missing assessments; of these, 107/133 (80%) were Grade 2+. These were managed with dose delays and dose reductions. The first Grade 2+ occurrence had resolved (recovered to Grade 1 or better) in 98/122 patients (80% of affected patients) by the data cutoff. Median time (range) to resolution of first Grade 2+ event was 68 (2–731) days for patients who received belantamab mafodotin. There were 24 patients treated with belantamab mafodotin who had unresolved Grade 2+ OEFs from their first event at their last visit; patients continued follow-up after OEFs per the clinical study protocol until disease progression, death, loss to follow-up, or the end of the study.

The DREAMM-3 final analysis suggests a consistent trend towards improvement in PFS with monotherapy belantamab mafodotin versus doublet pomalidomide-dexamethasone. DREAMM-3 did not meet the PFS endpoint at the primary analysis (data cutoff: September 12, 2022) as there was no statistically significant difference in PFS between the two treatment groups (HR [95% CI]: 1.03 [0.72, 1.47], *p* = 0.56). PFS HR showed stepwise improvements at the post-primary (0.90 [95% CI: 0.65–1.24]) and final analyses (0.87 [95% CI: 0.64, 1.18]); this incremental improvement over time is consistent with an immune-mediated response. At the primary analysis, median DOR was not reached (NR, 95% CI: 17.9 months, NR), indicating that responses were durable; with additional follow-up, median DOR was 25.3 months (95% CI: 20.7, 31.8) [7]. Median OS was 24.0 [95% CI:19.4, 33.2] months with belantamab mafodotin and 22.9 [95% CI: 16.0, 29.3] months with pomalidomide-dexamethasone (HR [95% CI]: 0.98 [0.73, 1.30]), showing no OS detriment.

The disease burden among patients who progressed within the first 2 months of treatment in DREAMM-3 was higher with belantamab mafodotin than with pomalidomide-dexamethasone in terms of ECOG PS, number of prior LOTs received, receipt of prior anti-CD38 therapy, sBCMA, and M-protein levels [7]. This imbalance in disease burden in the patients who progressed rapidly with belantamab mafodotin may account for the early crossing of the PFS curves.

Comparison of belantamab mafodotin monotherapy to doublet therapy may have been more appropriate as a test of a non-inferiority hypothesis over the superiority hypothesis tested in the DREAMM-3 trial design. Emerging evidence of an adaptive immune system response following belantamab mafodotin treatment suggests that longer follow-up prior to the primary analysis may have provided a better opportunity for the separation of efficacy endpoints in favor of belantamab mafodotin to be observed, although the non-proportional hazards effects would have remained in place [1, 7]. Aligning with adaptive immunity involvement, deep and durable responses are a hallmark of belantamab mafodotin treatment (observed with monotherapy and combination regimens), and led to improved PFS over time in this study [10–13]. The long median DOR for patients who responded to belantamab mafodotin, and the longer TBR for belantamab mafodotin compared to pomalidomide-dexamethasone, further support the rationale that longer readouts in clinical trials may be necessary for drugs that elicit immune responses [13]. Learnings from the DREAMM-3 trial and the increased understanding of the mechanism of action of belantamab mafodotin have been implemented into other ongoing trials [13].

No new safety signals were identified for belantamab mafodotin after long-term follow-up. Ocular events were manageable with dose reductions/delays. The rate of ocular AEs reported here is similar to previous reports on monotherapy and in combinations [14, 15]. In a previous subgroup analysis of patients who received long-term treatment (≥52 weeks) with belantamab mafodotin, most AEs were reported to have occurred at or before cycle 17 [16]. Despite the need for dose delays and/or reductions, clinical responses with belantamab mafodotin treatment were maintained or deepened in most cases, demonstrating that management of AEs using dose modifications did not impact clinical activity [16]. This was also demonstrated with the BVd and BPd combination regimens [14].

In conclusion, belantamab mafodotin monotherapy has clinical activity in patients with RRMM. Long-term treatment with belantamab mafodotin did not present additional safety signals, and ocular AEs were manageable with dose modifications/delays. BVd/BPd combination studies had higher efficacy and similar safety profiles.

## Supplementary information

Supplementary Material

## Data Availability

GSK makes available anonymized individual participant data and associated documents from interventional clinical studies that evaluate medicines, upon approval of proposals submitted to https://www.gsk-studyregister.com/en/.
