# Supplementary material for: Final analysis results from DREAMM-3, a phase 3 study of belantamab mafodotin versus pomalidomide plus dexamethasone in patients with relapsed/refractory multiple myeloma
Source: Blood Cancer J. 2026 Oct 17;16(1):184. doi: 10.1038/s41408-026-01646-0 (PMC13676618; doi:10.1038/s41408-026-01646-0)
Supplement: Supplementary file 1 [file 41408_2026_1646_MOESM1_ESM.pdf]

Supplementary materials

Methods

Study design

Patients received belantamab mafodotin 2.5 mg/kg intravenously every 3 weeks (Q3W), or oral pomalidomide 4 mg once daily (Days 1–21) plus oral dexamethasone (40 mg; 20 mg for patients aged >75 years) once weekly (Days 1, 8, 15, and 22) in a 28-day cycle until disease progression, death, unacceptable toxicity, withdrawal of consent, loss to follow-up, or end of study, whichever came first. Efficacy and safety assessments were performed Q3W for both arms. Patients in the belantamab mafodotin arm were prescribed prophylactic preservative-free artificial tears ≥4–8 times daily beginning on Cycle 1 Day 1 until end of treatment; frequency was increased up to every 2 hours in cases of ocular symptoms.

Belantamab mafodotin dose modifications were permitted to manage treatment-related toxicities. Following dose delays, belantamab mafodotin treatment could resume with or without dose reductions (to 1.9 mg/kg) after the toxicity resolved to Grade 1 or less. Dose modifications for pomalidomide/dexamethasone were permitted per drug labels.

Key patient inclusion criteria	Key patient exclusion criteria
• Confirmed diagnosis of MM according to IMWG criteria<br>• Age ≥18 years<br>• Measurable disease including at least one of serum-protein ≥5 g/L, urine M-protein ≥200 mg/24 hours or involved FLC level ≥100 mg/L with serum FLC ratio <0.26 or >1.65<br>• Autologous SCT >100 days prior, or transplant ineligible<br>• Received ≥2 prior lines of therapy, including ≥2 consecutive cycles of both lenalidomide and a proteasome inhibitor (given separately or in combination) and refractory to or progressing on or within 60 days of last line of treatment (or not achieving a	• Prior B-cell maturation antigen-targeted therapy or pomalidomide treatment<br>• Prior allogeneic SCT<br>• Treatment with an anti-MM monoclonal antibody <30 days prior to first study dose.<br>• Symptomatic amyloidosis, active POEMS syndrome, or plasma cell leukemia<br>• Systemic anti-myeloma therapy or investigational drug within <14 days or 5 half-lives of first study dose<br>• Active renal condition, unstable liver or biliary disease, active infection, or active bleeding<br>• Known human HIV infections, HBV, or HCV positive test result <3 months prior to first dose. Patients with positive hepatitis C antibody due to prior resolved

minimal response)<br>• ECOG PS 0–2<br>• Adequate organ system function<br>• Agreement to contraception guidelines, including agreement to applicable pregnancy prevention/controlled distribution program as indicated for pomalidomide	disease may be enrolled if a confirmatory negative hepatitis C RNA test is obtained<br>• Previous/concurrent malignancies other than MM, unless considered medically stable for >2 years<br>• Known reactivity to drugs chemically related to belantamab mafodotin, pomalidomide, dexamethasone, or any components of the study intervention

Independent Ethical Committees and Institutional Review Boards

**Name of IEC/IRB Committee and Address**
**Australia**
Sir Charles Gairdner Group Human Research Ethics Committee, Sir Charles Gairdner Hospital, Sir Charles Gairdner Hospital Avenue, First Floor, E Block, Nedlands, Western Australia, 6009, Australia
Barwon Health Human Research Ethics Committee, Level 2, Kitchener House, Geelong Hospital, Geelong, Victoria, 3220, Australia
Central Coast Local Health District, Level 1, Health Services Building, Gosford Hospital, Holden Street, Gosford, New South Wales, 3860, Australia
Royal Hobart Hospital, 48 Liverpool Street, Hobart, Tasmania, 7000, Australia
Central Northern Adelaide Health Service Ethics of Human Research Committtee, The Queen Elizabeth Hospital, Department of Haematology and Oncology, 28 Woodville Road, Woodville South, South Australia, 5011, Australia
South Western Sydney Local Health District, Locked Bag 7017, New South Wales, 1871, Australia
Monash Health Human Research Ethics Committee, Level 2, I Block, Monash Medical Centre, Clayton, Victoria, 3168, Australia
Saint Vincent's Hospital (Melbourne) HREC D (RGO), Research Governance Unit, Level 5, Mary Aikenhead Building, 27 Victoria Parade, Fitzroy, Victoria, 3065, Australia
Western Health, 176 Furlong Road, Level 3, Western Centre for Health Research and Education, Saint Albans, Victoria, 3021
Northern Sydney Local Health District, Executive Unit, Building 51, Level 1, Southern Campus, Royal North Shore Hospital, Pacific Highway, Saint Leonards, New South Wales, 2065, Australia
**Belgium**
Ethisch Comité OLV (Onze-Lieve-Vrouwziekenhuis) Ziekenhuis vzw<br>Ethisch Comité, Moorselbaan 164, Onze-Lieve-Vrouwziekenhuis - Campus Aalst, Aalst, 9300, Belgium
**Brazil**
Comitê de Ética em Pesquisa da Fundação Antônio - AC Camargo Câncer Center, Rua Professor Antônio Prudente, 211, Liberdade, São Paulo, 01509-900, Brazil
Comitê De Ética Em Pesquisa do Hospital Mãe de Deus, José de Alencar n 286, 11 floor, Porto Alegre, 90880-481, Brazil
Comitê De Ética Em Pesquisa do Hospital de Clínicas de Porto Alegre, Rua Ramiro Barcelos, 2350 – 2 º andar - Sala 2227, Porto Alegre, 90035-007, Brazil
Comitê De Ética Em Pesquisa do Hospital Israelita Albert Einstein / SP, Avenida Albert Einstein, 627/701 - 2º subsolo - Bloco A, Morumbi, São Paulo, 05651-901, Brazil

Name of IEC/IRB Committee and Address
Comitê De Ética Em Pesquisa Invitare, Rua Itaipu, 475, São Paulo, 04052-010, Brazil
Comitê De Ética Em Pesquisa do Hospital Pró-Cardíaco/RJ, Rua Voluntários da Pátria, 435 - 8º andar, Botafogo, Rio de Janeiro, 22270-005, Brazil
Comitê De Ética Em Pesquisa da Real Benemérita Associaçao Portuguesa de Beneficência - Hospital São Joaquim, Rua Maestro Cardim, 769, Bela Vista, São Paulo, 01323-900, Brazil
Comitê De Ética Em Pesquisa do Instituto do Câncer do Ceará, Rua Papi Júnior, 1222, 6 º andar - Rodolfo Teófilo, Fortaleza, 60430-230, Brazil
Comitê De Ética Em Pesquisa do Hospital Pró-Cardíaco/RJ, Rua Voluntários da Pátria, 435 - 8º andar, Botafogo, Rio de Janeiro, 22270-005, Brazil
Comitê de Ética em Pesquisa da Fundação Antônio - AC Camargo Câncer Center, Rua Professor Antônio Prudente, 211, Liberdade, São Paulo, 01509-900, Brazil
**Bulgaria**
Republic of Bulgaria Ministry of Health Ethic Committee for Clinical Trials, 8, Damyan Gruev Street, Sofia, 1303, Bulgaria
**Canada**
242277 | Health Research Ethics Board of Alberta - Cancer Committee (HREBA-CC), 10104-103 Avenue NW, Suite 1500, Edmonton, Alberta, T5J 4A7, Canada
**China**
EC of The Affiliated Hospital of Xuzhou Medical University, No.99 West Huaihai Road, Xuzhou, 221000, China
Shenzhen 2nd People's Hospital, No3002 Sungang West Rd, Futian District, Gaoming District Foshan, Shenzhen, 518035, China
EC of Henan Cancer Hospital, No 127. Dongming Road, Zhengzhou, 450008, China
EC of The First Hospital of Jilin University, No. 1 Xinmin Street, 2nd Floor, GCP Central Pharmacy 1, Changsha, 130021, China
245752 | Peking University Third Hospital Medical Science Research Ethics Committee, No. 49, Huayuan North Road, Haidian District, Beijing, 100191, China
EC of the First Affiliated Hospital of Nanchang University, No.17 Yongwaizheng Street, Nanchang City, Jiangxi, 330006, China
245935 | EC of Peking Union Medical College Hospital, No.1 Shuaifuyuan, 1F, Translational Medicine Building, Dongcheng District, Beijing, 100730
EC of Beijing Hospital, No.1 Dahua Road, Dongdan, Dongcheng District, Beijing, 100730, China
EC of The First Affiliated Hospital, Med. School, No.79 Qingchun Rd., Hangzhou, 310003, China
Clinical Trial Ethics Committee of West China Hospital, No. 37 Guoxue Alley, West China Hospital, Sichuan University, Chengdu, Sichuan, 610041, China
EC of Tianjin Medical University Cancer Institute&Hospital, Huan'hu West Road, He'xi District, Tianjin, 300060, China
EC of Beijing Friendship Hospital Capital Medical University, NO 95 Yong An Road, Beijing, 100050, China
EC of Guangdong General Hospital, No.106 Zhongshan Second Road, Yuexiu District, Guangzhou, 510080, China
EC of Blood Diseases Hospital Chinese Academy of Medical Sciences, No.288 Nanjing Road, Heping District, Tianjin, 300020, China
**France**
Comité de Protection des Personnes, 46 rue des Saints-Pères, Paris, 75006, France
Comité de Protection des Personnes Sud-Est II, 59 Boulevard Pinel, Bâtiment Pinel, Bron, 69500, France
Comité de Protection des Personnes, 46 rue des Saints-Pères, Paris, 75006, France

Name of IEC/IRB Committee and Address
**Germany**
Ethik-Kommission der Bayerischen Landesärztekammer, Mühlbaurstraße 16, Muenchen, 81677, Germany
**Greece**
National Organization for Medicines, 284 Mesogion Ave, Cholargos, 15562, Greece
**Hungary**
Egeszsegugyi Tudomanyos Tanacs Klinikai Farmakologiai Etikai Bizottsaga, Báthory u. 10, Budapest, 1054, Hungary
**Italy**
Azienda Ospedaliero Universitaria Consorziale Policlinico di Bari, Piazza Giulio Cesare,11, Bari, 70124, Italy
**Japan**
Matsuyama Red Cross Hospital Institutional Review Board, Bunkyo-cho 1, Matsuyama-shi, 790-8524, Japan
Hokkaido University Hospital Institutional Review Board, North 14, West 5, Kita-ku, Sapporo-shi, Hokkaido, 060-8648, Japan
The University of Osaka Hospital Institutional Review Board, 2-15, Yamadaoka, Suita-city, Osaka, 565-0871, Japan
Nagoya City University Hospital Institutional Review Board, Aza Kawasumi 1, Mizuho-ku Mizuho-cho, Aichi-Ken, 467-8602, Japan
Tohoku University Hospital Institutional Review Board, 1-1, Seiryo-machi, Aoba-ku, Sendai-city, Miyagi, 980-8574, Japan
Ogaki Municipal Hospital Institutional Review Board, 4-86 Minaminokawa-cho, Ogaki-city, Gifu, 503-8502, Japan
University Hospital, Kyoto Prefectural University of Medicine Institutional Review Board, Kamigyo-ku Kawaramachidori Hirokoji Agaru Kajii-cho 465, Kyoto-shi, 602-8566, Japan
National Hospital Organisation Shibukawa Medical Center Institutional Review Board, Shiroi 383, Shibukawa-city, Gunma, 377-0280, Japan
The Institute of Medical Science The University of Tokyo Hospital Institutional Review Board, Shirokanedai 4-6-1, Minato-ku, Tokyo, 108-8639, Japan
Jikei University Hospital Institutional Review Board, 3-19-18, Nishi-Shimbashi, Minato-ku, Tokyo, 105-8471, Japan
Japan Community Health Care Organisation Kyoto Kuramaguchi Medical Center Institutional Review Board, 27, Kita-ku Koyamashimofusa-cho, Kyoto-shi, Kyoto, 603-8151, Japan
Japanese Red Cross Medical Center Institutional Review Board, Hiroo 4-1-22, Shibuya-ku, Tokyo, 150-8935, Japan
**Korea, Republic of**
Seoul National University Bundang Hospital, 172, Dolma-ro, Bundang-gu, Institutional Review Board, 13th floor, Healthcare Innovation Park, Seongnam-si, 13605, Korea, Republic of
Institutional Review Board of Seoul National University Hospital, 172, Dolma-ro, Bundang-gu, Institutional Review Board, 3th floor, Healthcare Innovation Park, Seongnam-si, 13605, Korea, Republic of
Institutional Review Board of National Cancer Center, 323 Ilsan-ro, Ilsandong-gu, Goyang-si, Gyeonggi-do, National Cancer Center, Gyeonggi-do, 10408, Korea, Republic of
Institutional Review Board of Gachon University Gil Medical Center, 21 Namdong-daero 774beon-gil, Namdong-gu, Gachon University Gil Medical Center, Incheon, 21565, Korea, Republic of
Institutional Review Board of Chonnam National University Hwasun Hospital, 322, Seoyang-ro, Hwasun-eup, Hwasun-gun, Jeollanam-do, 58128, Korea, Republic of

Name of IEC/IRB Committee and Address
Institutional Review Board of The Catholic University of Korea, Seoul St. Mary's Hospital, 222, Banpo-daero, Seocho-gu, Seoul, 06591, Korea, Republic of
Institutional Review Board of Asan Medical Center, Building 88, 2nd Floor, Convergence Research Building, Olympic-ro 43-gil, Songpa-gu, Asan Life Science Research, Seoul, 05505, Korea, Republic of
**Netherlands**
Stichting Beoordeling Ethiek Biomedisch Onderzoek, Weiersstraat 1C, METC Stitching BEBO, Assen, 9401, Netherlands
**Poland**
Niezalezna Komisja Bioetyczna, Building No.1,3rd floor, room 3.26, Medycznym , Debinki 7, Gdańsk, 80-211, Poland
**Russian Federation**
Local Ethics Committee at SBIH of Kaluga Region "Kaluga Regional Clinical Hospital", Vishnevskogo street, 1, Kaluga, 248007, Russian Federation
LEC at SBEI HPE "Samara State Medical University" of the MoH of the RF, 20, Gagarina street, Samara, 443079, Russian Federation
Local Ethics Committee at SBHI of Novosibirsk Region "SNR Clinical Hospital", 130, Nemirovicha Danchenko street, Novosibirsk, 630087, Russian Federation
SRI of Hematology and Transfusiology, 16, 2nd Sovetskaya street, Russian Hematology and Transfusiology Research Center, Saint-Petersburg, 191024, Russian Federation
Interuniversity Ethics Committee, 8, Trubestskaya street, building 1, Moscow, 119991, Russian Federation
Sverdlovsk Regional Clinical Hospital, 185, Volgogradskaya Street, Ekaterinburg, 620102, Russian Federation
Territorial Clinical Hospital, 3-A, Partizana Zhelesnyaka street, Krasnoyarsk, 660022, Russian Federation
Local Ethics Committee Federal State Budgetary Healthcare Institution Federal Medical Research Center, 2, Accuratova street, Saint-Petersburg, 197341, Russian Federation
Local Ethics Committee of SHI Komi Republican Oncology Dispenser, 46, Niuvchimskoe shosse, Syktyvkar, 167904, Russian Federation
Kirov SR Institute of Hematology and Blood Transfusion FMBA, 72, Krasnoarmeyskaya street, Kirov, 610027, Russian Federation
State healthcare facility Tula Regional Hospital, 1A, Yablochkova street, Tula, 300053, Russian Federation
LEC at LLC "MEDIS", Tropinina street, 4a, Nizhny Novgorod, 603137, Russian Federation
**Spain**
CEIM Hospital General Universitario Gregorio Marañon, Pabellon de Gobierno, C/ Dr. Esquerdo 46, Fundación para la Investigación Biomédica, H. Gragorio Marañon, Madrid, 28007, Spain
**United Kingdom**
Research & Development Imperial College Healthcare National Health Service Trust<br>Imperial College Healthcare National Health Service Trust, Joint Research Compliance Office, Charing Cross Hospital, Fulham Palace Road, London, W6 8RF, United Kingdom
Research & Development - Plymouth Hospitals National Health Service Trust, Derriford Road, Plymouth, PL6 8DH, United Kingdom
Research & Development - University Hospital of North Staffordshire National Health Service Trust, Trust Headquarters, City General Site, A Block, Newcastle Road, Stoke-on-Trent, ST4 6QG, United Kingdom

Name of IEC/IRB Committee and Address
Research & Development Oxford University Hospitals National Health Service Trust, Old Road, Oxford Cancer Centre, 2nd Floor Administration Office, Headington, Oxfordshire, OX3 7LE, United Kingdom
National Research Ethics Service Committee - East of Scotland research ethics committee 2 Ninewells Hospital and Medical School, Terrestrial Animal Health Standards Commission Research & Development Office, Residency Block, Level 3, Dundee, DD1 9SY, United Kingdom
Research & Development - Barts Health National Health Service Trust, Barts Health National Health Service Trust, 5 Walden Street, Joint Research Management Office, London, Central Region, E1 2EF, United Kingdom
National Health Service Lothian, Research & Development, Western General Hospital, Crewe Road, Edinburgh, Lothian Region, EH4 2XU, United Kingdom
National Health Service Lanarkshire, Monkscourt Avenue, Airdrie, Lanarkshire, ML6 0JS, United Kingdom
Research & Development Nottingham University Hospitals National Health Service Trust Research & Development Nottingam University Hospitals, National Health Service Trust, C Floor, South Block, Queens Medical Centre Campus, Nottingham, NG7UTH, United Kingdom
**United States**
United States Oncology, Incorporation Institutional Review Board, 10101 Woodloch Forest, The Woodlands, Texas, 77380, United States

Supplementary Table 1: Summary of additional safety findings where AEs affected ≥10% of patients

	Belantamab mafodotin (N=217)	Pomalidomide-dexamethasone (N=102)
**Investigations, any event, n (%)**	109 (50)	41 (40)
Platelet count decreased	34 (16)	13 (13)
Aspartate aminotransferase increased	36 (17)	6 (6)
Blood lactate dehydrogenase increased	35 (16)	1 (<1)
Gamma-glutamyltransferase increased	30 (14)	6 (6)
Neutrophil count decreased	20 (9)	16 (16)
Alanine aminotransferase increased	19 (9)	10 (10)
White blood cell count decreased	14 (6)	11 (11)
**General disorders and administrations site conditions, any event, n (%)**	80 (37)	37 (36)
Pyrexia	45 (21)	14 (14)
Fatigue	23 (11)	15 (15)
Edema peripheral	8 (4)	12 (12)
**Gastrointestinal disorders, any event, n (%)**	81 (37)	34 (33)
Diarrhea	29 (13)	12 (12)
Nausea	25 (12)	5 (5)
Constipation	13 (6)	10 (10)
**Musculoskeletal and connective tissue disorders, any event, n (%)**	64 (29)	33 (32)
Back pain	17 (8)	10 (10)

Abnormal hepatic function was reported in 13 (6%) of patients treated with belantamab mafodotin and no patients treated with pomalidomide-dexamethasone.

Supplementary Figure 1: Study design

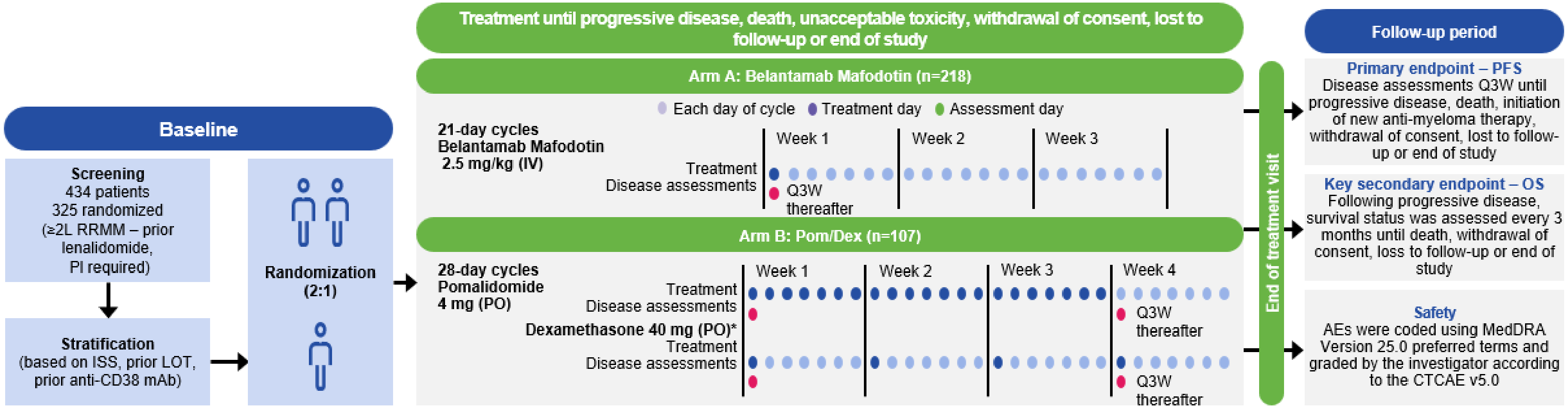

*Dexamethasone dosage for those >75 years (20 mg). Study design figure *©2024 International Myeloma Society. Reused with permission. This figure was included in a poster that was previously presented at the 2024 IMS Annual Meeting. All rights reserved.*

2L, second-line; AEs, adverse events; CTCAE, Common Terminology Criteria for Adverse Events;ISS, international staging system; IV, intravenous; LOT, lines of therapy; mAb, monoclonal antibody; MedDRA, Medical Dictionary for Regulatory Activities; Q3W, every three weeks; MM, multiple myeloma; OS, overall survival; PFS, progression-free survival; PO, by mouth; RRMM, relapsed/refractory multiple myeloma

Supplementary Figure 2: Kaplan-Meier plots of OS (A), DOR (B), and PFS2 (C)

A)

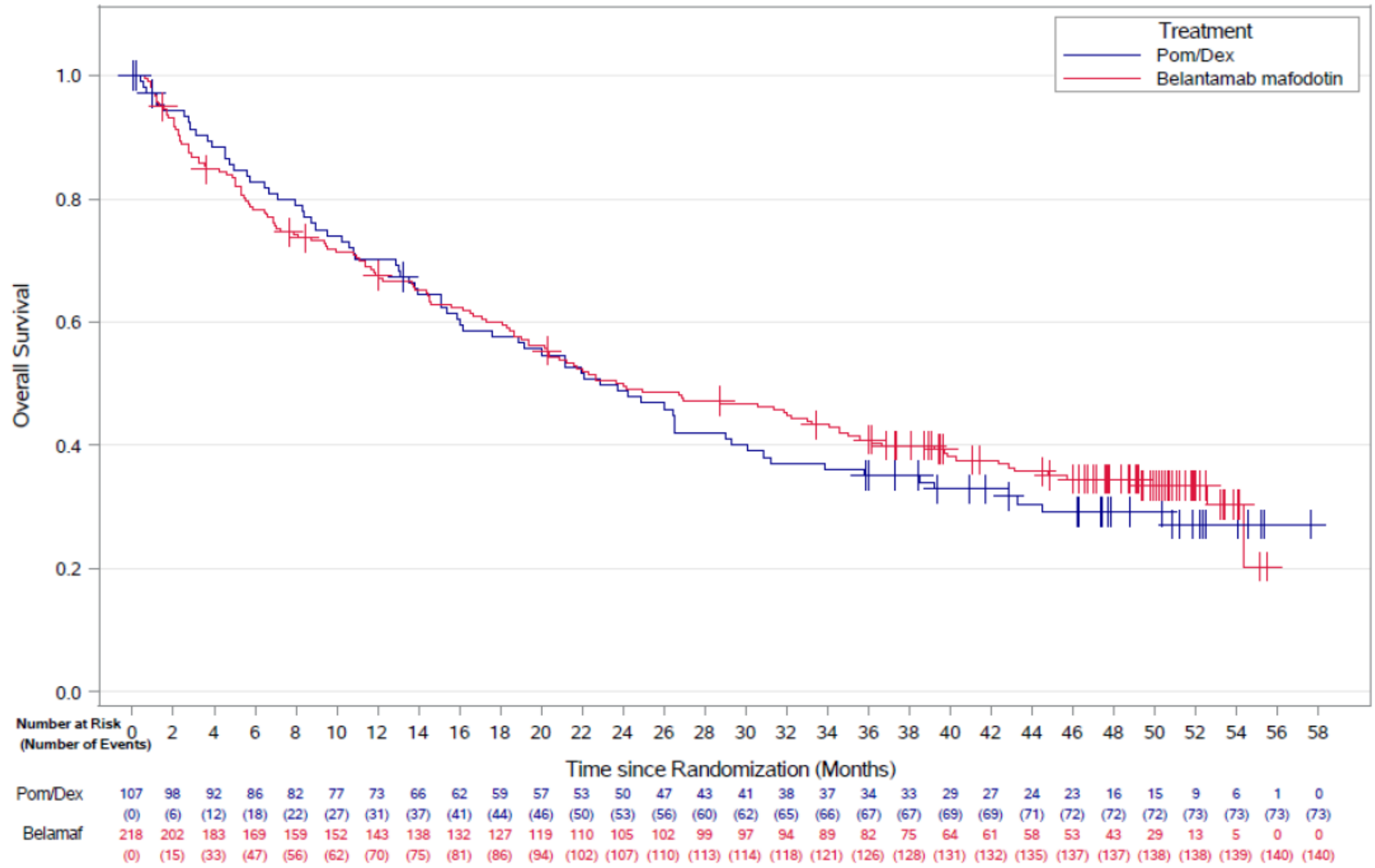

B)

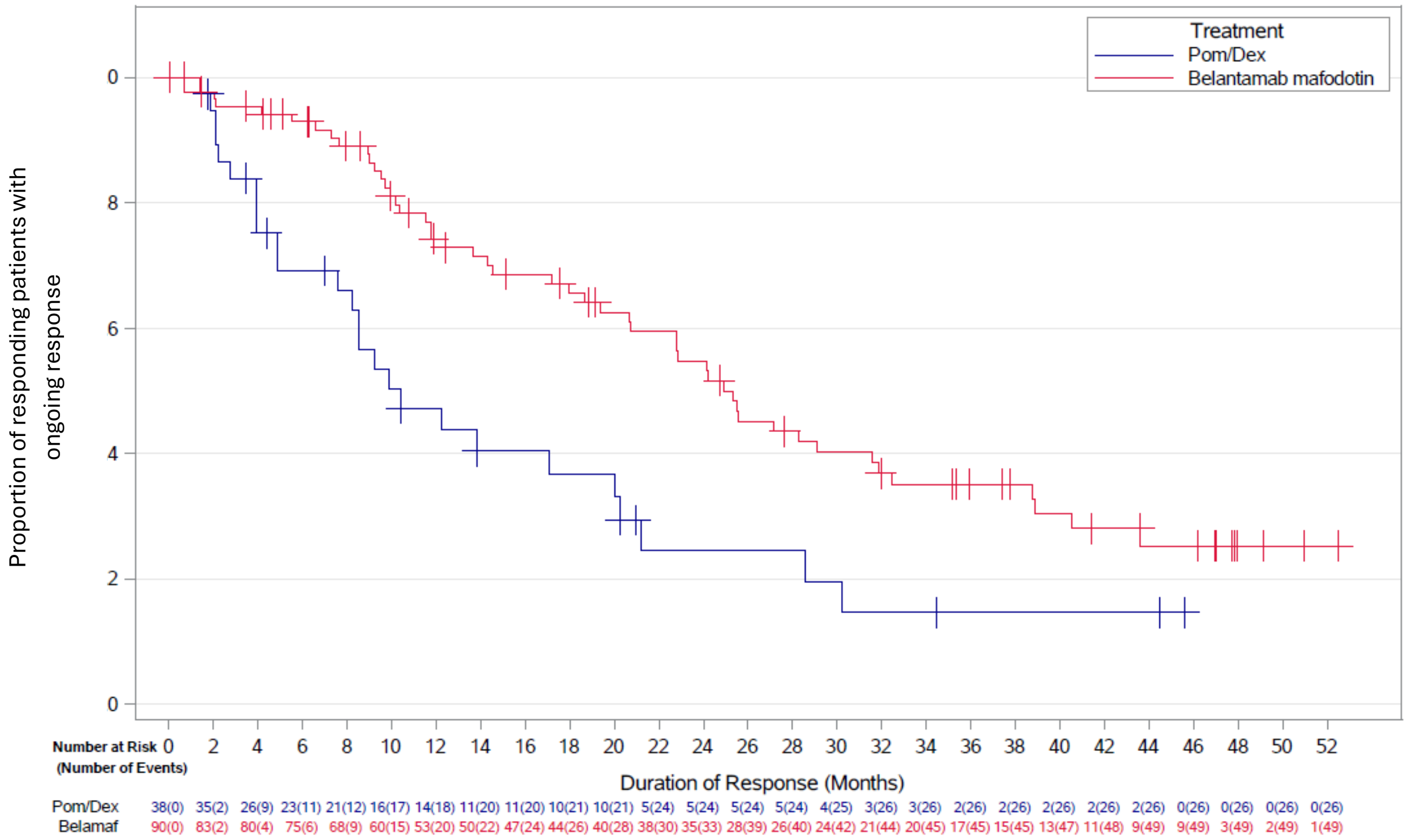

C)

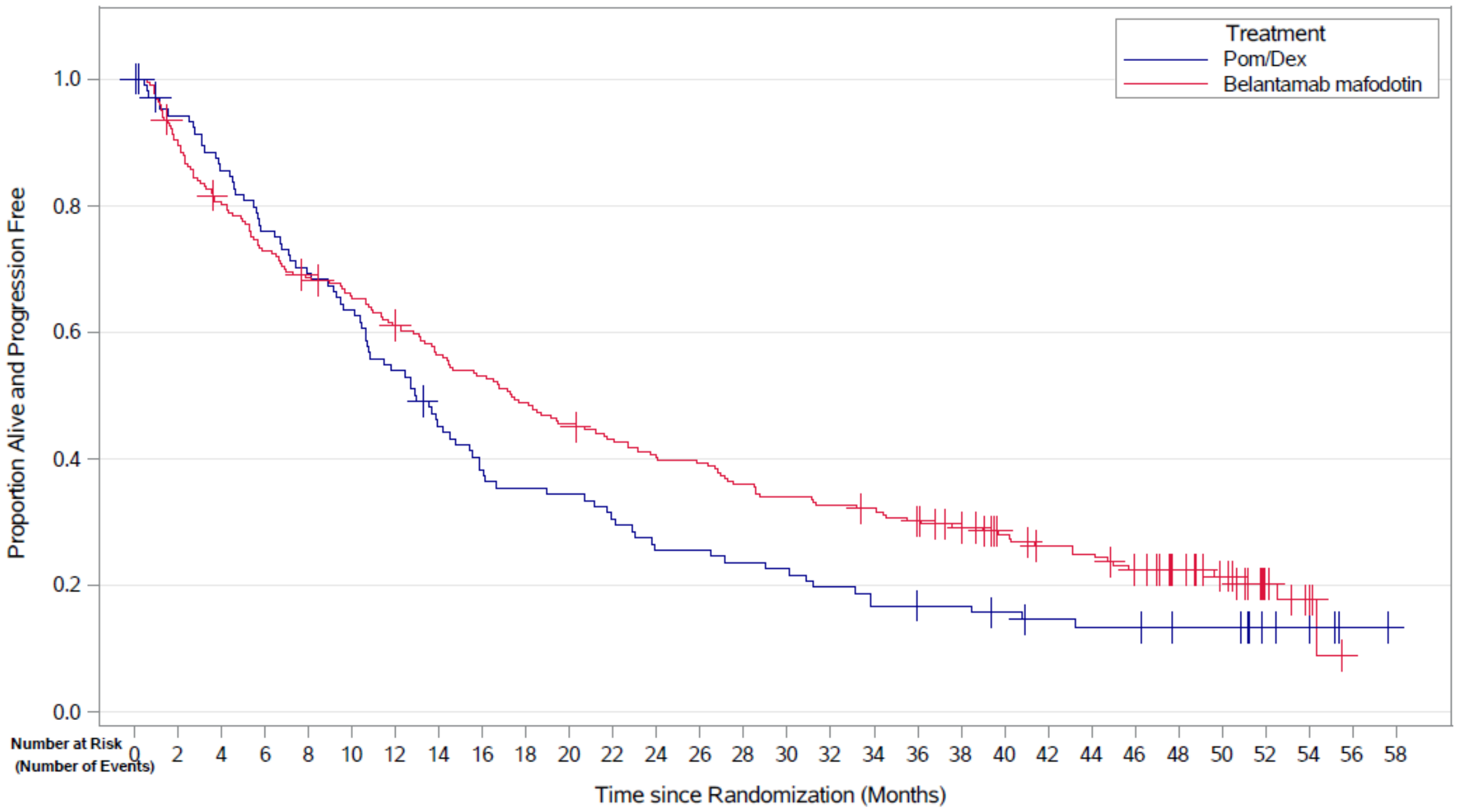

Number at Risk (Number of Events)	0	2	4	6	8	10	12	14	16	18	20	22	24	26	28	30	32	34	36	38	40	42	44	46	48	50	52	54	56	58
Pom/Dex	107	98	89	79	72	66	56	46	39	36	35	31	26	26	24	23	20	17	16	16	14	12	11	11	9	9	5	4	1	0
	(0)	(6)	(15)	(25)	(32)	(38)	(48)	(57)	(64)	(67)	(68)	(72)	(77)	(77)	(79)	(80)	(83)	(86)	(86)	(86)	(87)	(88)	(89)	(89)	(89)	(89)	(89)	(89)	(89)	(89)
Belamaf	218	196	173	157	147	139	129	119	112	103	96	90	84	82	75	71	68	66	61	55	47	42	40	34	28	20	9	4	0	0
	(0)	(21)	(43)	(59)	(68)	(75)	(84)	(94)	(101)	(110)	(117)	(122)	(128)	(130)	(137)	(141)	(144)	(145)	(149)	(151)	(153)	(156)	(158)	(162)	(162)	(163)	(164)	(165)	(166)	(166)
